# Supplementary figures and images for: Changes in cell morphology and function induced by the NRAS Q61R mutation in lymphatic endothelial cells
Source: PLoS One. 2024 May 29;19(5):e0289187. doi: 10.1371/journal.pone.0289187 (PMC11135733; doi:10.1371/journal.pone.0289187)

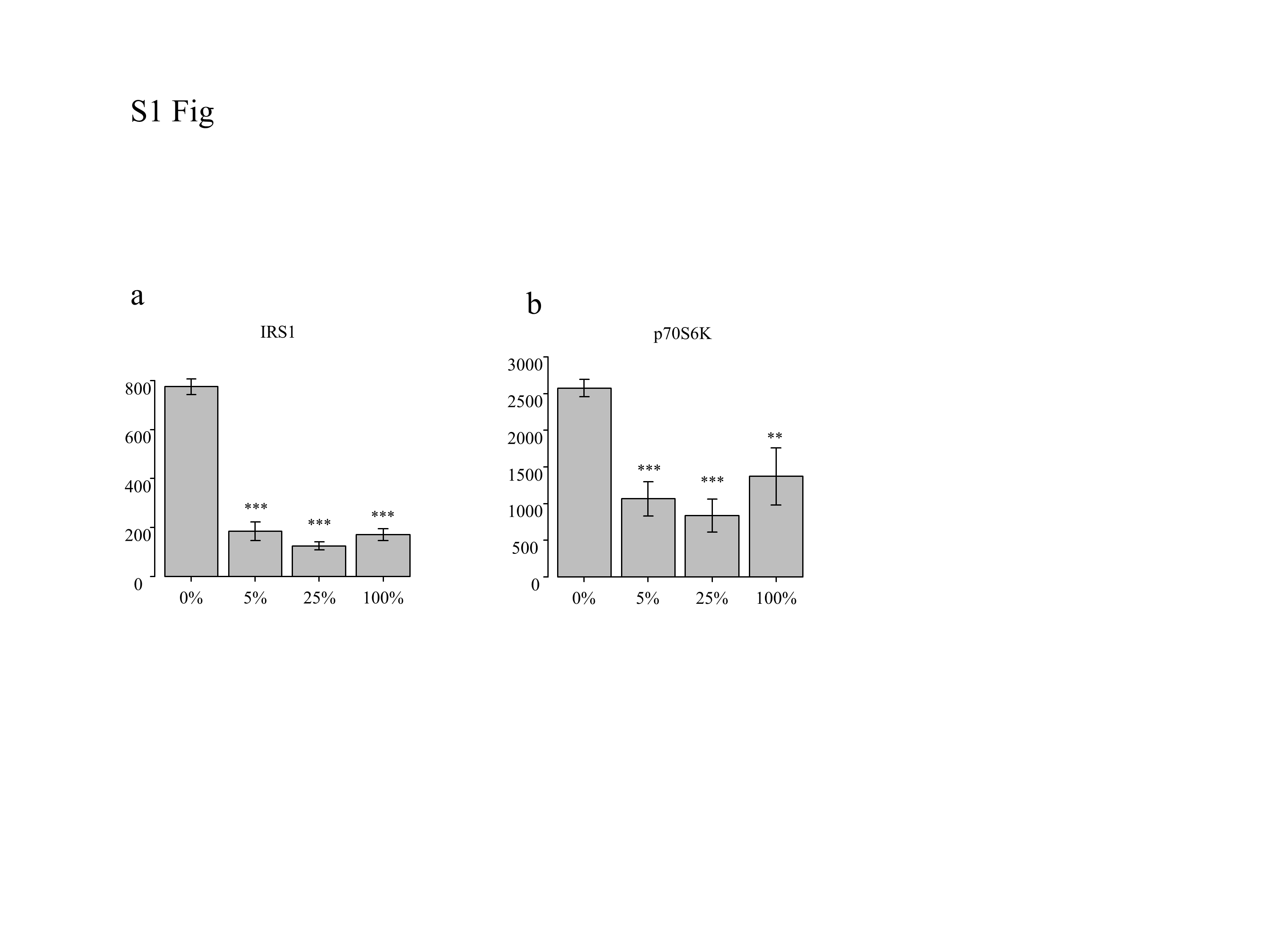

Supplement: S1 Fig — Bars represent mean ± SD from triplicate wells. *p<0.05, **p<0.01, ***p<0.001, compared with 0%. (TIF) [file pone.0289187.s003.tif]

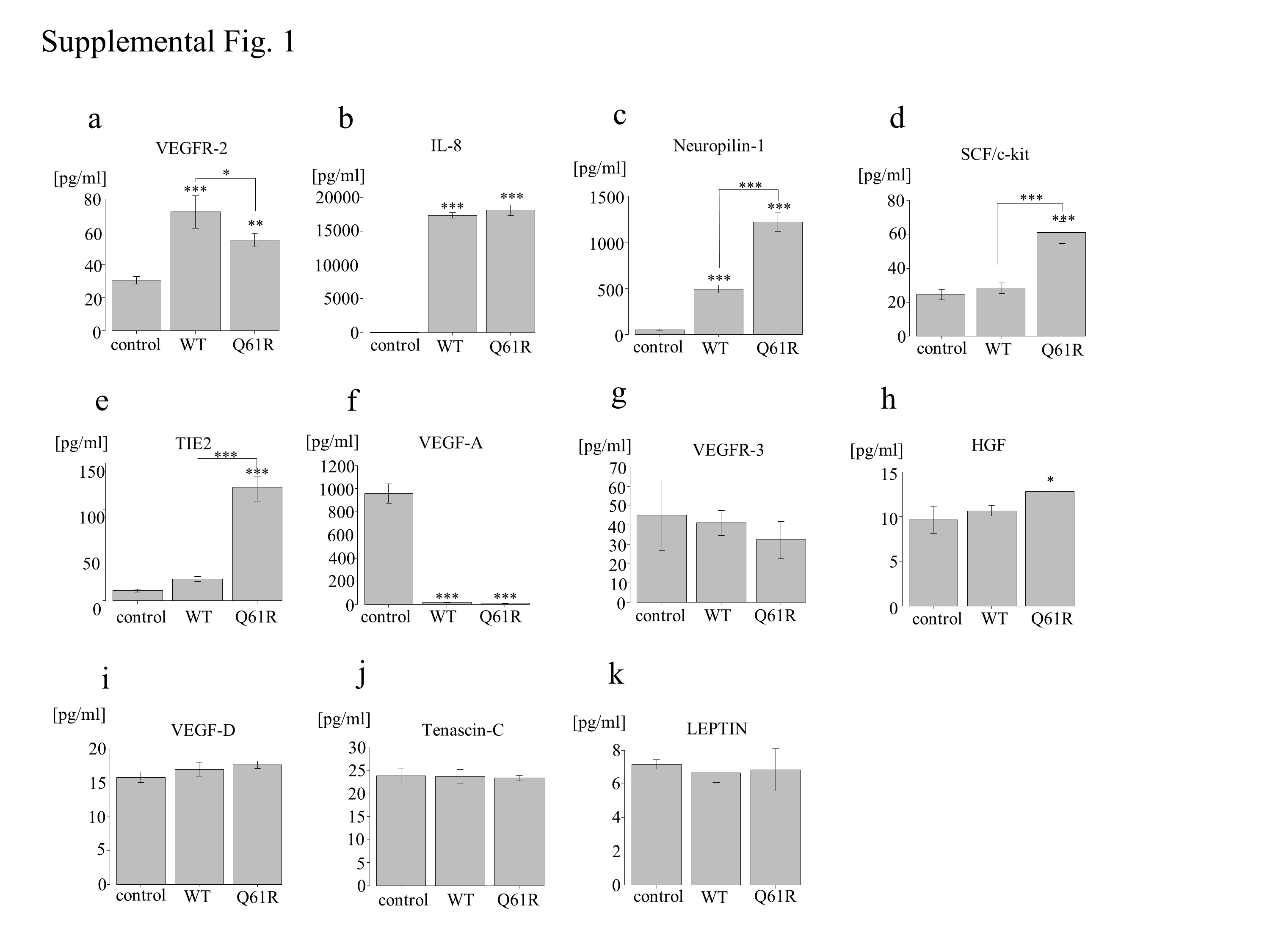

Supplement: S2 Fig — Vascular endothelial growth factor receptor (VEGFR)-2 (a), interleukin (IL)-8 (b), neuroplin-1 (c), stem cell factor (SCF)/c-kit (d), TIE2 (e), vascular endothelial growth factor (VEGF)-A (f), VEGFR-3 (g), hepatocyte growth factor (HGF) (h), VEGF-D (i), tenascin-C (j), and leptin (k). Bars represent mean ± SD from triplicate wells. *p<0.05, **p<0.01, ***p<0.001, compared with control. NRASQ61R and NRASWT HDLECs were also compared. (TIF) [file pone.0289187.s004.TIF]

AKT

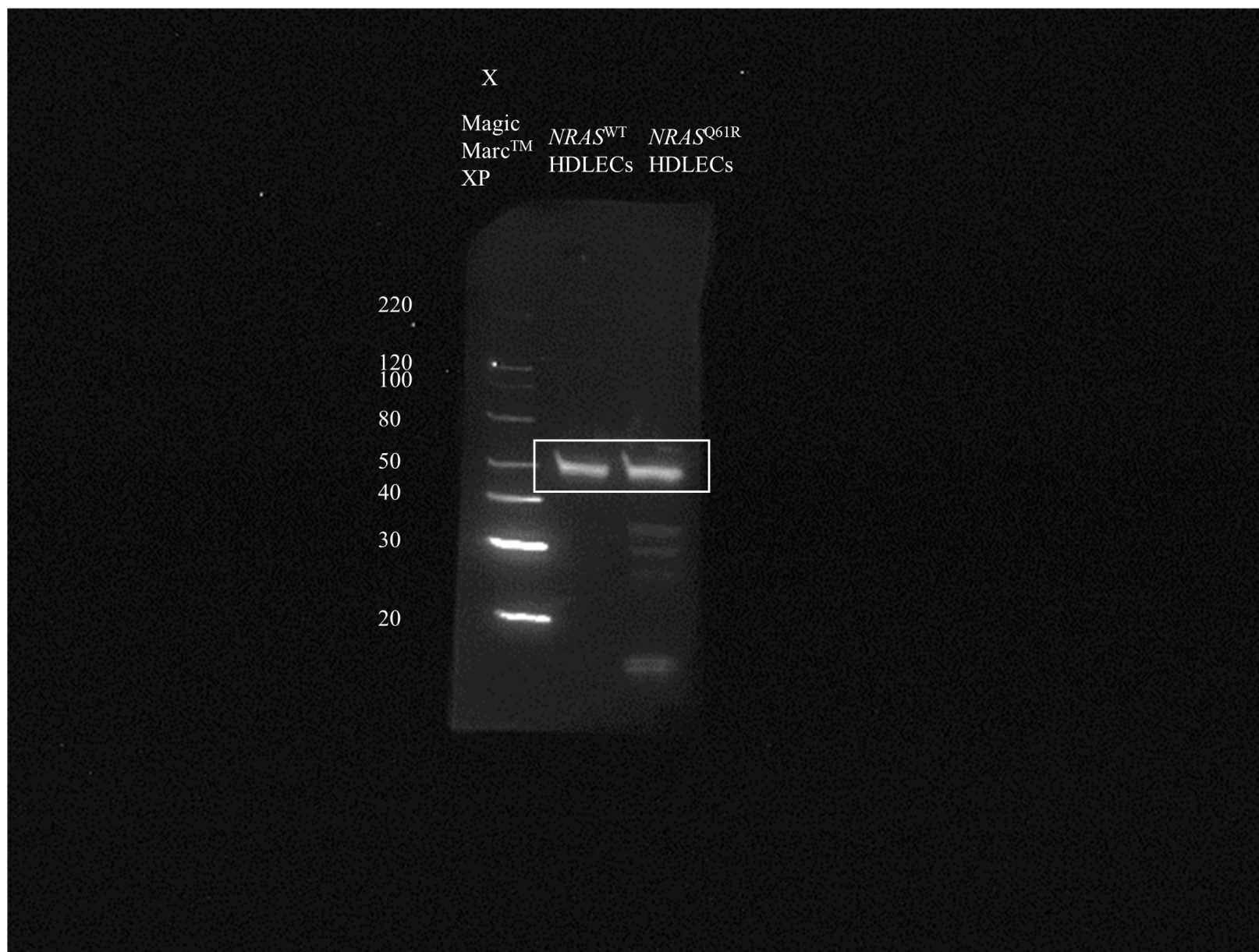

Supplement: S1 Raw image — (PDF) [file pone.0289187.s005.pdf]

p-AKT

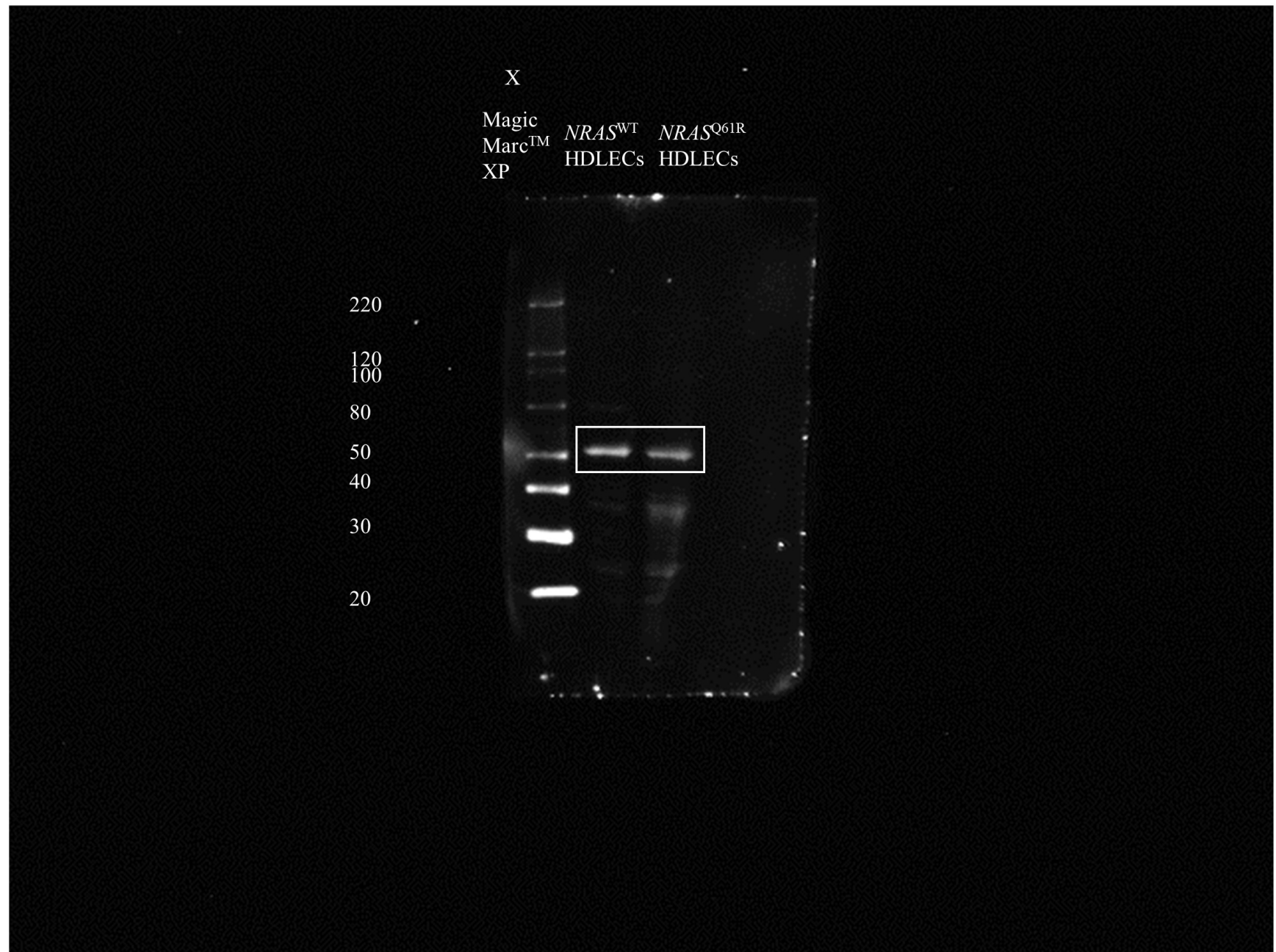

Supplement: S2 Raw image — (PDF) [file pone.0289187.s006.pdf]

ERK1/2

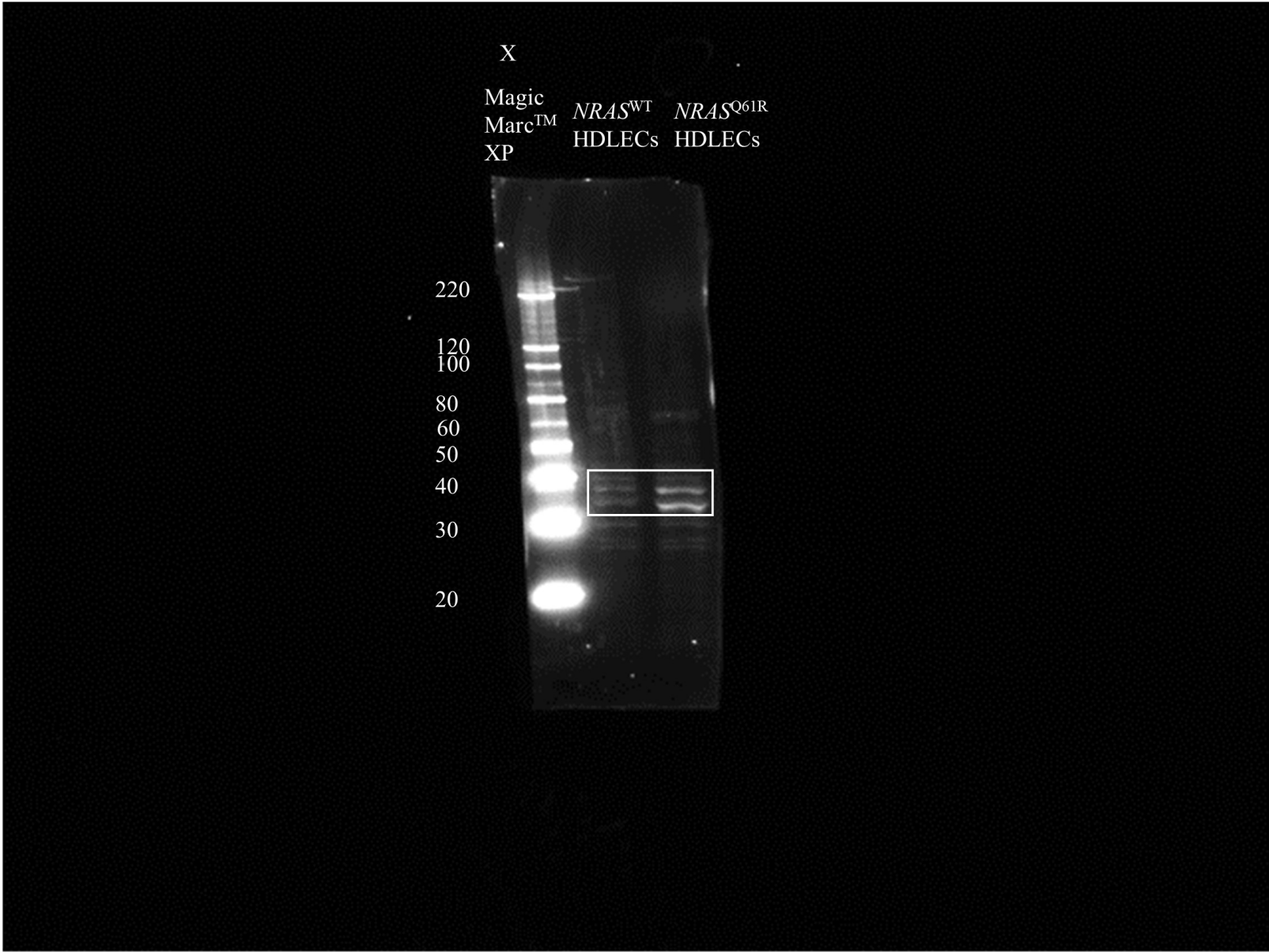

Supplement: S3 Raw image — (PDF) [file pone.0289187.s007.pdf]

p-ERK1/2

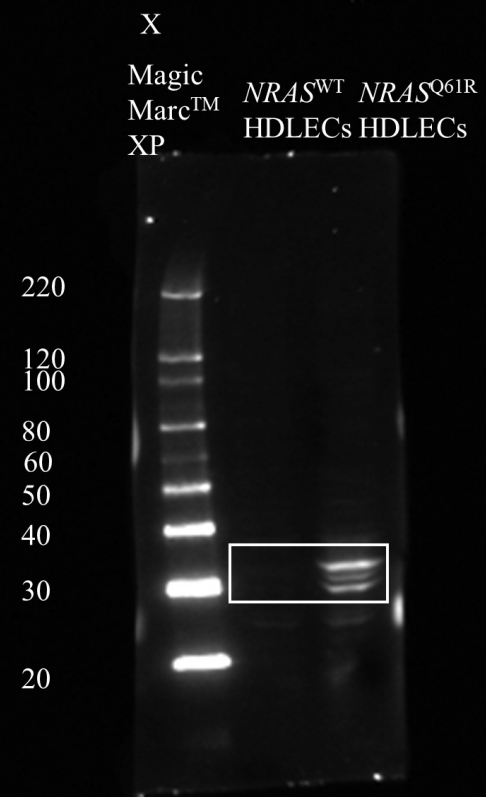

Supplement: S4 Raw image — (PDF) [file pone.0289187.s008.pdf]

GAPDH

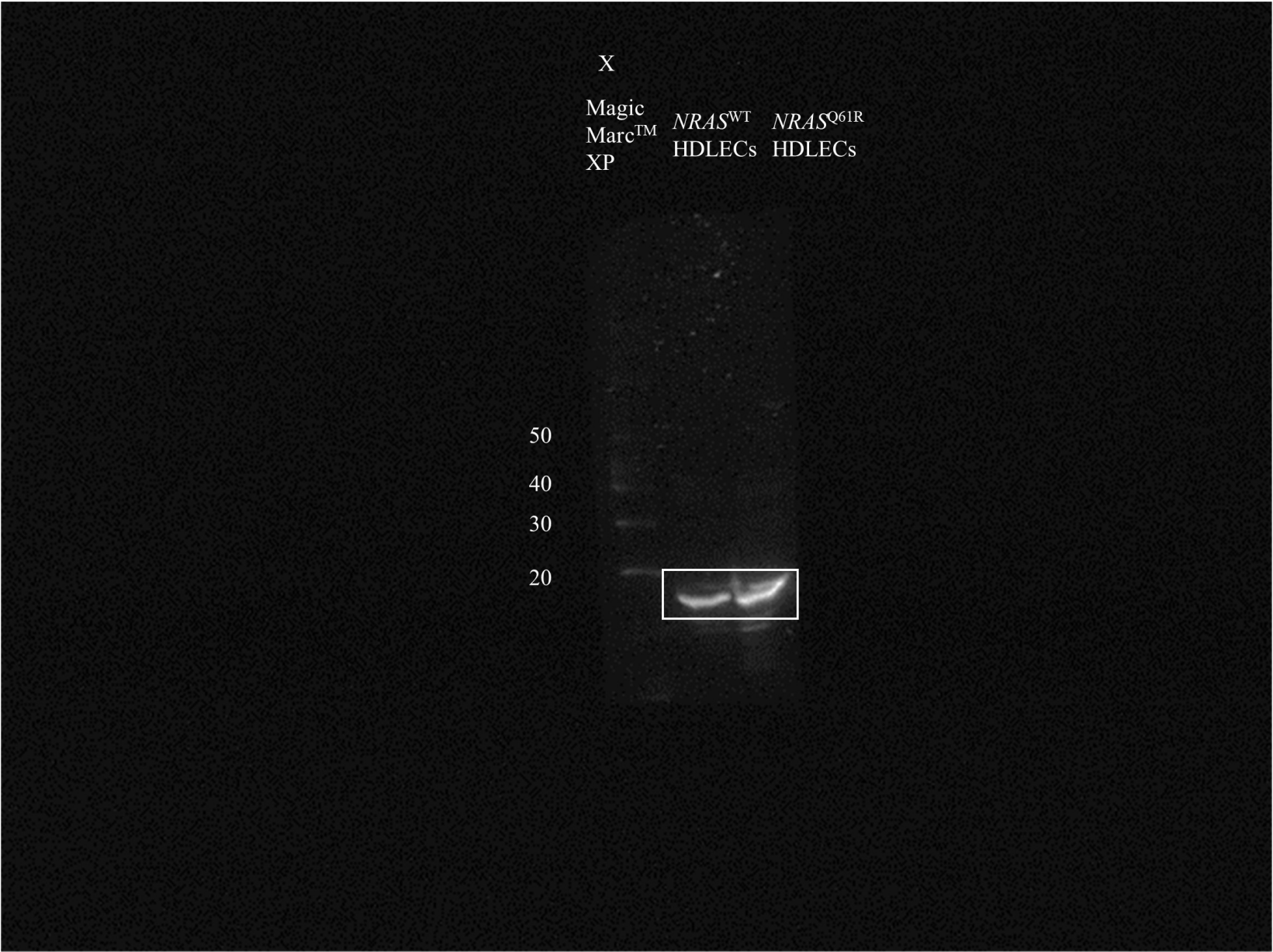

Supplement: S5 Raw image — (PDF) [file pone.0289187.s009.pdf]
