# Supplementary material for: Changes in cell morphology and function induced by the NRAS Q61R mutation in lymphatic endothelial cells
Source: PLoS One. 2024 May 29;19(5):e0289187. doi: 10.1371/journal.pone.0289187 (PMC11135733; doi:10.1371/journal.pone.0289187)
Supplement: Attachment — Submitted filename: Responce_to_reviewer.docx [file pone.0289187.s010.docx]

PONE-D-23-21611

Title: **Changes in cell morphology and function induced by the *NRAS* Q61R mutation in** **lymphatic endothelial cells**

**Responses to Journal Requirements**

*Journal Requirements:*

*When submitting your revision, we need you to address these additional requirements.*

*1. Please ensure that your manuscript meets PLOS ONE's style requirements, including those for file naming. The PLOS ONE style templates can be found at　https://journals.plos.org/plosone/s/file?id=wjVg/PLOSOne_formatting_sample_main_body.pdf and　https://journals.plos.org/plosone/s/file?id=ba62/PLOSOne_formatting_sample_title_authors_affiliations.pdf*

Thank you for this comment. We ensured that the revised manuscript complied with the style requirements of PLOS ONE, including those for file naming. We referred to the provided style templates for guidance.

*2. We suggest you thoroughly copyedit your manuscript for language usage, spelling, and grammar. If you do not know anyone who can help you do this, you may wish to consider employing a professional scientific editing service. Whilst you may use any professional scientific editing service of your choice, PLOS has partnered with both American Journal Experts (AJE) and Editage to provide discounted services to PLOS authors. Both organizations have experience helping authors meet PLOS guidelines and can provide language editing, translation, manuscript formatting, and figure formatting to ensure your manuscript meets our submission guidelines. To take advantage of our partnership with AJE, visit the AJE website (http://learn.aje.com/plos/) for a 15% discount off AJE services. To take advantage of our partnership with Editage, visit the Editage website (www.editage.com) and enter referral code PLOSEDIT for a 15% discount off Editage services.  If the PLOS editorial team finds any language issues in text that either AJE or Editage has edited, the service provider will re-edit the text for free.*

*Upon resubmission, please provide the following:*

a. *The name of the colleague or the details of the professional service that edited your manuscript.*
b. *A copy of your manuscript showing your changes by either highlighting them or using track changes (uploaded as a *supporting information* file).*
c. *A clean copy of the edited manuscript (uploaded as the new *manuscript* file).*

Thank you for your guidance on enhancing the manuscript’s language quality. We would like to inform you that for the previous submission, the manuscript was professionally copyedited by H. Nikki March, PhD, from Edanz (https://jp.edanz.com/ac). Following your recent suggestions, we have engaged their services again to ensure the manuscript adheres to the guidelines of PLOS ONE.

Upon resubmission, we will provide the edited manuscript with changes highlighted, as well as a clean copy of the revised text. We appreciate the discounts offered through your partnerships with AJE and Editage, and we will consider them for future reference.

*3. Thank you for stating the following in the Acknowledgments Section of your manuscript:*

*“..The present study was supported in part by a Practical Research Project for Rare/Intractable Diseases (JP22ek0109515) from Japan’s Agency for Medical Research and Development, AMED. Finally, we thank H. Nikki March, PhD, from Edanz (https://jp.edanz.com/ac) for editing a draft of this manuscript.”*

*We note that you have provided funding information that is not currently declared in your Funding Statement. However, funding information should not appear in the Acknowledgments section or other areas of your manuscript. We will only publish funding information present in the Funding Statement section of the online submission form.*

*Please remove any funding-related text from the manuscript and let us know how you would like to update your Funding Statement. Currently, your Funding Statement reads as follows:*

*“The authors received no specific funding for this work.”*

*Please include your amended statements within your cover letter; we will change the online submission form on your behalf.*

Thank you for your instructions regarding the placement of funding information in our manuscript. We have removed the funding-related text from the Acknowledgments section of the manuscript as per your guidance. In the cover letter accompanying our resubmission, we have included the corrected Funding Statement. The revised statement accurately reflects the financial support we received for our research. We kindly request that the changes be reflected in the online submission form to align with the updated manuscript.

The amended Funding Statement is as follows: "The present study was supported in part by a Practical Research Project for Rare/Intractable Diseases (JP22ek0109515) from Japan’s Agency for Medical Research and Development, AMED."

We trust that this meets the requirements for the Funding Statement section and appreciate your assistance in updating our submission records.

*4.  We note that you have stated that you will provide repository information for your data at acceptance. Should your manuscript be accepted for publication, we will hold it until you provide the relevant accession numbers or DOIs necessary to access your data. If you wish to make changes to your Data Availability statement, please describe these changes in your cover letter and we will update your Data Availability statement to reflect the information you provide.*

In our previous submission, we indicated that we would provide repository information for our data. However, we would like to update the Data Availability statement to read as follows:

"Raw data were generated at Gifu University Hospital. Derived data supporting the findings of this study are available from the corresponding author Michio Ozeki on request."

We apologize for any inconvenience this change may cause and appreciate your assistance in updating our submission to reflect this new information.

*5. PLOS ONE now requires that authors provide the original uncropped and unadjusted images underlying all blot or gel results reported in a submission’s figures or Supporting Information files. This policy and the journal’s other requirements for blot/gel reporting and figure preparation are described in detail at https://journals.plos.org/plosone/s/figures#loc-blot-and-gel-reporting-requirements and https://journals.plos.org/plosone/s/figures#loc-preparing-figures-from-image-files. When you submit your revised manuscript, please ensure that your figures adhere fully to these guidelines and provide the original underlying images for all blot or gel data reported in your submission. See the following link for instructions on providing the original image data:* <https://journals.plos.org/plosone/s/figures#loc-original-images-for-blots-and-gels>*.　In your cover letter, please note whether your blot/gel image data are in Supporting Information or posted at a public data repository, provide the repository URL if relevant, and provide specific details as to which raw blot/gel images, if any, are not available. Email us at plosone@plos.org if you have any questions.*

As per the instructions provided, we will submit the original uncropped and unadjusted images for all blot or gel results reported in the figures or Supporting Information files. We have reviewed the requirements of PLOS ONE for blot/gel reporting and figure preparation, and we will ensure that our figures fully adhere to these guidelines.

Thank you for directing us to the relevant resources. The original image data will be provided upon submission of our revised manuscript.

**Responses to reviewer #1’s comments**

*Reviewer #1: The manuscript, “Changes in cell morphology and function induced by NRAS Q61R mutation in lymphatic endothelial cells” presents the morphological characterization and functional profiling of human dermal lymphocyte endothelial cells (LECs) upon transfection with lentivirus vector carrying specific NRAS mutation. The work includes protein expression analysis, and metabolomic profiling upon such transfection. According to this in vitro investigation NRAS Q61R human dermal LECs have poor tube formation and high cell proliferation and migration ability with increasing ratios of mutated cells. They demonstrate the inactivation of the PIK3/AKT/mTOR pathway and hyperactivation of the RAS/MAPK/ER pathway in the transfected cells. The latter is inhibited by trametinib, which targets MAP kinase. The study provides a possible mechanism for Kaposis lymphangiomatosis pathology and suggests the potential treatment strategies.*

*The study is well designed and presented in a coherent manner. There are a few presentation and language concerns*:

We thank the reviewer for their helpful comments regarding our manuscript. Our responses to the reviewer’s comments are shown below. Changes in the text of the revised manuscript are shown in red font.

*Presentation Concerns:
i. In the Materials and Methods section under the heading “Gene transfection of HDLEC cell line using lentiviruses”, the authors have written “NRASQ61R expression in these cells was checked by polymerase chain reaction (PCR).” Probably they mean insertion rather than expression. In case of expression profiling “real time PCR” must have been performed. The authors may provide details as supplementary material.*

We thank the reviewer for the comment and agree with their comment. We revised “expression” to “insertion” (P6, L87). We have provided sequence results as supplementary material.

*ii. Figure 1: The magnification scale bar needs to be provided.*

We thank the reviewer for the comment and have inserted a scale bar in Fig. 1a-c.

*iii. Images (a) and (b) in Figure 1 need to be more clear to draw conclusions in the text.*

We thank the reviewer for the comment. We replaced Fig. 1a and b with a fluorescence version, which is clearer.

*iv. Under Metabolomics of HDLECs heading on page no. 26, last paragraph, Fig. 5. Is written instead of Fig. 6.*

Thank you for bringing to our attention the incorrect figure call-out in our manuscript. In response, we have carefully reviewed the manuscript and amended the text accordingly. The phrase "Fig. 5" has now been corrected to "Fig. 6" (P21, L366).

*Language suggestions:
The manuscript may be revised for some minor language corrections for the purpose of clarity and precision. For instance:
i. In the abstract, “MEK” abbreviation is not explained.*

Thank you for pointing out the unexplained abbreviation of "MEK" in the Abstract of our manuscript. In response to your feedback, we have revised the term “MEK inhibitor” in the Abstract to read “MAPK kinase (MEK) inhibitor”, aligning with the usage and definition provided in the main text (P2, L23-24).

*ii. In the Introduction, it’s unclear what authors mean by “KLA is a subtype of GLA … [and presents with] poorer outcomes than GLA patients”. Do they actually mean “worse outcomes than GLA patients with other subtypes”?*

Thank you for your comment regarding the clarity of our description of Kaposiform lymphangiomatosis (KLA) in the Introduction. We revised this sentence to “KLA is characterized by spindle-shaped lymphatic endothelial cells (LECs) and presents with more serious symptoms (abnormal coagulation, thrombocytopenia, and hemorrhagic pericardial and pleural effusion) and a worse prognosis than GLA” (P3, L32-34)

*iii. In the Methods section under the heading “Gene transfection of HDLEC cell line using lentiviruses”, the authors have written “The recombinant lentiviral solution was diluted with EBM2 to give solutions with initial concentrations of 1:4 to 1:10.” May be the authors should consider “prepare solutions with initial concentrations of 1:4 to 1:10”.*

Thank you for your suggestion regarding the phrasing in the Methods section. We revised the sentence to: “We prepared the recombinant lentiviral solutions at initial concentrations of 1:4 to 1:10” (P5, L81-82).

**Responses to reviewer #2’s comments**

*The manuscript by Yasue et al. describes the morphological and functional alterations observed in juvenile foreskin-derived human dermal lymphatic endothelial cells (HDLECs) after stable lentiviral insertion of a mutated form of the NRAS gene (Q61R variant). The Authors argue that somatic low-level mutation of the NRAS gene in HDLECs could explain the lymphatic abnormalities observed in patients with kaposiform lymphangiomatosis (KLA).
However, several concerns weaken the reliability of this work. The main concerns relate to the consistency and presentation of the data. Furthermore, the analysis presented is strictly descriptive, functional experiments are required to support the claimed correlation and causality. Previously published papers report the NRASQ61R variant found in KLA samples, with no evidence as to which cell type is mutated. The Authors propose that the NRASQ61R mutation of HDLECs is capable of inducing KLA-like aberrations based on similar molecular and morphological abnormalities observed in vitro. At the same time, they suggest that low-frequency mutated cells could affect the phenotype of distant cells even regardless of their mutational status, thus raising questions about the ability to induce the same phenotype on HDLECs by RAS mutation in other cell types in the microenvironment. To clarify this point, single cell RNA sequencing of KLA samples or coculture of WT HDLECs with NRAS mutant stromal cells should be performed.*

Thank you for your insightful review and constructive feedback on our manuscript. We deeply appreciate your understanding of the content and the positive evaluation you have given. Your valuable advice has significantly contributed to improving the quality of our work.

We agree with your well-reasoned points, particularly regarding the consistency and presentation of the data. We have made the necessary revisions to address these concerns. Furthermore, your suggestion about the *NRAS* gene mutation in lymphatic endothelial cells is insightful. We hypothesized that the *NRAS* mutation occurs in these cells, leading to the morphological and molecular biological changes observed, which in turn cause structural abnormalities in the lymphatic vessels. This hypothesis was supported by reproducible results, indicating that low-frequency mosaic mutations in cells might impact surrounding cells within the microenvironment.

As you rightly pointed out, conducting the single-cell RNA sequencing of KLA samples or coculture of WT HDLECs with *NRAS* mutant stromal cells might provide valuable insights into how mutated cells induce molecular and genetic changes in non-mutated cells. We recognize the importance of this analysis and consider it a future research direction. Once again, we are grateful for your thorough review and valuable suggestions, which will undoubtedly strengthen our study.

We have added the following sentences to the Discussion: “Our study suggests that the abnormal proliferation and morphological anomalies of lymphatic tissues within systemic lesions of patients with KLA are caused by the *NRAS^Q61R^* mutation in LECs. Furthermore, our data suggest this is triggered by low-frequency somatic mosaic mutations. However, there were several limitations in this study. The first is that, although mutated cells may influence surrounding cells, further evidence for this is not provided. The relationship between growth factors and the etiology of vascular anomalies is not understood; however, growth factors secreted by some *NRAS^Q61R^* HDLECs within the lesion may affect the proliferation of mutated cells and abnormalities in peripheral normal LECs. To investigate this further, additional functional analysis experiments will be necessary. Moreover, the single-cell RNA sequencing of KLA samples or co-culturing *NRAS* mutant stromal cells with *NRAS^WT^* HDLECs might be a valuable approach. Such analysis might unravel the complex molecular and genetic interactions induced by mutated cells within a heterogeneous cell population, offering deeper insights into the cellular mechanisms underpinning KLA. The second limitation concerns the pharmacological effects of trametinib. In Fig 3C, the number of live cells after trametinib treatment was the same for all samples, regardless of the percentage of *NRAS^Q61R^* cells present. This suggests the presence of factors other than those involved in RAS/MAPK/ERK pathway activation affected the drug’s efficacy. The dosage of trametinib, regardless of mutation status, may have exerted a therapeutic effect on all LECs. Further investigation is needed on these points. Lastly, although cell density was carefully adjusted to be uniform in all wells during sample preparation, the baseline cell density between WT and mutant cells appears different in the time-lapse photography. This discrepancy is attributed to the inherent size differences between the cell types. The mutant cells are naturally larger than the WT cells, which might give the impression of a higher or uneven density, even with an equivalent number of cells. The perceived difference in density is likely due to cell size variation, not an actual difference in cell numbers.” (P26, L454-P28, L476)

*MAJOR
- Intensive improvement of the image quality in the figures is required to appreciate the morphology of the cells and structures*

*- The WT cells in most of the figures are not visible as they are not colored. This is especially inconvenient when mixed cultures are being analyzed and NRASQ61R cells are the only observable, with no WT cell information in the dark areas. Red staining should be performed on WT cells to better understand the cell-cell interactions and morphological changes.*

Thank you for your valuable feedback regarding the visualization of WT cells in our figures. We understand your concern about the lack of color contrast in the images, particularly when analyzing mixed cultures where only *NRAS*^Q61R^ cells are visible. We considered your suggestion to use red staining for WT cells to highlight cell-cell interactions and morphological changes more distinctly. However, we decided against this approach due to concerns that introducing a red stain might impart some chemical effects on the cells, which might have led to different phenomena than those currently observed, thus affecting the integrity of our results. Instead, to address your feedback, we prepared images of *NRAS*^WT^ and *NRAS^Q61R^* HDLECs with fluorescence, which provides significantly improved quality (Fig. 1a, 1b). These enhanced images provide a clearer visualization of cells, ensuring that the morphology and interactions are more easily discernible. We hope this modification adequately addresses your concerns and allows for a more thorough understanding of our findings.

*- The time-lapse photography in supplementary files a, b, allows observation of WT and mutant cells, however cell density at baseline appears to be different. Can you explain this discrepancy?*
Thank you for your observation regarding the cell density in our time-lapse photography. When preparing the samples, we meticulously adjusted the cell density to ensure uniformity across all wells, specifically targeting a density of 1 × 10^4^ cells/well in a 96-well plate. However, as you rightly noted, there appears to be a discrepancy in the baseline cell density between the WT and mutant cells. We think this perceived difference in density may be attributed to the inherent size variation between the cell types. The mutant cells are naturally larger compared with WT cells, which might give the impression of a higher density or uneven distribution, even when the actual cell numbers are equivalent. This variation in cell size, rather than an actual difference in cell density, likely accounts for the observations noted in the time-lapse images.

We hope this explanation resolves any concerns regarding the consistency of our experimental setup and the subsequent interpretation of our results.

We have added the following sentences to the Discussion: “Lastly, although cell density was carefully adjusted to be uniform in all wells during sample preparation, the baseline cell density between WT and mutant cells appears different in the time-lapse photography. This discrepancy is attributed to the inherent size differences between the cell types. The mutant cells are naturally larger than the WT cells, which might give the impression of a higher or uneven density, even with an equivalent number of cells. The perceived difference in density is likely due to cell size variation, not an actual difference in cell numbers." (P27, L471-P28, L476)

*- The results of the proliferation assay should be better clarified: an increase in proliferation in NRASQ61R cells is reported in the abstract and discussion, while the opposite phenotype is described in the results, in figure 3C (decreased in NRASQ61R cells) and in the conclusion.*

Thank you for highlighting the inconsistency in our manuscript regarding the proliferation of *NRAS*^Q61R^ cells. We appreciate your careful reading and the opportunity to correct this error. Upon reviewing your comment and revisiting our data, we agree that there was a discrepancy between the Results section and the Abstract. The results presented in Figure 3C and discussed in the Results section, which indicate decreased proliferation in *NRAS*^Q61R^ cells, are accurate. It was an oversight on our part to report otherwise in the Abstract.

To rectify this, we have amended the Abstract to accurately reflect the experimental findings. We revised “*NRAS*^Q61R^ human dermal LECs showed poor tube formation and high cell proliferation and migration ability with increasing ratios of mutated cells.” to “*NRAS*^Q61R^ human dermal lymphatic endothelial cells showed poor tube formation, a low proliferation rate, and high migration ability with an increase in the ratio of mutated cells.” in the Abstract (P2, L20-22). This change aligns the Abstract with the detailed results and conclusions of our study, ensuring consistency and clarity throughout the manuscript.

*- The same consideration for the number of nets formed in NRASQ61R cells with respect to WT: the result described in the text is opposite with respect to the graph of figure 2B. Please clarify this point.*

Thank you for your observation regarding the discrepancy between the text and the graph presented in Figure 2B of our manuscript. As mentioned, the description in the text was incorrect. We revised the manuscript and the corrected sentence now accurately reflects the data shown in Figure 2B. We revised “The number of nets of 0% *NRAS*^Q61R^ was significantly lower than that of 5%, 25%, and 100% *NRAS*^Q61R^” to “The number of nets of 0% *NRAS^Q61R^* was significantly higher than those of 5%, 25%, and 100% *NRAS*^Q61R^” (P14, L237-238).

*- In figure 3C the amount of live cells after trametinib treatment is the same in all samples, regardless of the percentage of NRASQ61R cells; how can this apparent independence of drug efficacy from ERK MAPK activation be explained?*

Thank you for your comments. It is intriguing that the quantity of live cells in Figure 3C remained unchanged after trametinib treatment across all samples, regardless of the percentage of *NRAS*^Q61R^ cells present. This observation raises questions about the potential involvement of factors beyond the activation of the RAS/MAPK/ERK pathway in influencing the efficacy of trametinib. Moreover, this consistency in treatment response, irrespective of mutation status, may suggest that the dosage of trametinib had a therapeutic effect on all lymphatic endothelial cells. However, to gain a comprehensive understanding of these phenomena, further in-depth investigations are required. These findings underscore the complexity of trametinib’s mechanisms of action and highlight the need for additional research to elucidate the underlying factors contributing to its effects on lymphatic endothelial cells. Although these observations are insightful, it is important to note that they may not fully address all the questions raised, and more extensive research is warranted to provide comprehensive answers.

In response to the observation regarding Figure 3C, we have added this as a limitation in the Discussion section of our manuscript. We have included the following statement: " The second limitation concerns the pharmacological effects of trametinib. In Fig 3C, the number of live cells after trametinib treatment was the same for all samples, regardless of the percentage of *NRAS*^Q61R^ cells present. This suggests the presence of factors other than those involved in RAS/MAPK/ERK pathway activation affected the drug’s efficacy. The dosage of trametinib, regardless of mutation status, may have exerted a therapeutic effect on all LECs. Further investigation is needed on these points. " (P27, L466-471).

*- Analysis of the metabolome of NRASQ61R cells revealed increased secretion of ANG-2 and decreased VEGFR3 compared with WT cells. The authors suggested that hypersecretion of ANG-2 by NRASQ61R HDLECs may cause decrease in VEGFR-3 levels, thereby imparing tube formation. Functional experiments based on loss or gain of function are needed to strenghten this claim.*

Thank you for your comments. As you rightly pointed out, conducting functional experiments to provide evidence for the claim that the hypersecretion of ANG-2 by *NRAS*^Q61R^ HDLECs leads to a decrease in VEGFR-3 levels is a crucial next step. This experimental validation will not only strengthen our hypothesis but also enhance the overall credibility of our findings. We appreciate your suggestion, and we fully acknowledge the importance of performing these functional experiments to further elucidate the mechanisms involved.

In response to the feedback on our findings related to the analysis of *NRAS*^Q61R^ cells, we have discussed this as a limitation in the Discussion of the revised manuscript as follows: “Our study suggests that the abnormal proliferation and morphological anomalies of lymphatic tissues occurring within systemic lesions in patients with KLA are caused by the *NRAS*^Q61R^ mutation in LECs. Furthermore, it indicates that this is triggered by low-frequency somatic mosaic mutations. However, there are several limitations to this experiment. The first is that while it is suggested that mutated cells may influence surrounding cells, further evidence for this was not provided. The relationship between growth factors and the etiology of vascular anomalies is not understood, but growth factors secreted by some *NRAS*^Q61R^ HDLECs within the lesion may not only affect the proliferation of mutated cells but also the abnormalities in peripheral normal LECs. To elucidate this, additional functional analysis experiments would be necessary.” (P26, L454-P27, L462).

*MINOR
- The titles in the results section are more suitable for materials and methods; please replace them with short sentences that briefly describe the result obtained.*

Thank you for your comments. In response to your suggestion, we have replaced the titles in the Results section with concise sentences that succinctly describe the key findings of each subsection. We revised the titles in the Results section as follows.

***“NRAS^Q61R^* HDLEC characteristics”** to **“*****NRAS*^Q61R^ HDLECs are large and have irregular-shaped morphology**” (P13, L213-214)

“**Tube formation assay*”*** to ***“*****Poor tube formation in *NRAS*^Q61R^ HDLECs is improved by inhibition of the mTOR/MEK pathway*”*** (P14, L227-228)

***“*Scratch assay*”*** to ***“NRAS*^Q61R^ HDLECs have a higher migration ability than *NRAS*^WT^ HDLECs**” (P16, L269-270)

***“*Cell** **proliferation assay*”*** to ***“*Increased proportion of mutant cells decreases the cell proliferation rate*”*** (P17, L283-284)

**“Analysis of signaling pathways in HDLECs by western blotting and multiplex protein assay”** to **“Hyperactivation of the RAS pathway in *NRAS*^Q61R^ HDLECs is improved by a MEK inhibitor”** (P18, L297-298)

**“Suspension array”** to ***“*Increased secretion of ANG-2 and decreased secretion of VEGFR3 from *NRAS*^Q61R^ HDLECs**” (p20, L332-333)

***“Metabolomics of HDLECs”*** to **“****The metabolome is altered in *NRAS*^Q61R^ HDLECs*”*** (P21, L353)

*- Figures 4B-d and 4B-e are mentioned in the text but missing from the figure.*

These call-outs were inadvertently retained in the text. To address this, we decided to include the data for Figure 4B-a and 4B-d in the Supplemental figures section. We apologize for any confusion caused by this oversight and appreciate your guidance in maintaining the integrity and completeness of our manuscript.

*- Please specify in figure 3B what it refers to: the legend of the figure refers to the proliferation data, while the text shows the space ratio result and the title on the y axis is missing.*

Thank you for your comment regarding Figure 3B in our manuscript. We acknowledge this discrepancy. The legend previously titled “Cell proliferation assay of *NRAS^Q61R^* and *NRAS*^WT^ HDLEC mixtures” has been changed to “The space ratio of the wound area at 0, 6, 12, 18, and 24 h.”(P16, L279-P17, L280). Additionally, we have added the title “space ratio” to the y-axis of Figure 3B to clarify what is being measured and presented. These modifications ensure that the figure, its legend, and the associated text in the manuscript are consistent and provide a clear understanding of the results presented.

*-in the last paragraph of the results chapter “Analysis of signaling pathways in HDLECs by western blotting and multiplex protein assay” there are some refuses to correct: AKT was described in the previous paragraph, so it shoud be something different; moreover, only the efficacy of trametinib is described while two P-values are reported which raise doubts about the described target.*

Thank you for your comment. We have reevaluated the data, and considering the importance of Akt, mTOR, and ERK, we have designated them as Fig. 4C-a, b, and c, respectively, and IRS-1 and p70s6k have been added to the Supplementary information (S1 Fig a and b). We understand that there was confusion regarding the figure numbering.

We revised these sentences in the Results to: “Additionally, to quantitatively measure protein expression levels, we performed multiplex protein assay. The median fluorescence intensity (MFI) of IRS-1 decreased significantly even in small amounts of *NRAS*^Q61R^ HDLEC mixtures (p<0.001, p<0.001, and p<0.001, respectively) (S1 Fig a). The MFI of AKT, the major protein of the PIK3 pathway, was decreased in the cell group containing 5% and 25% *NRAS*^Q61R^ (p=0.021 and p=0.007, respectively), but there was no statistically significant difference between that of *NRAS*^WT^ and *NRAS*^Q61R^ (Fig 4B-a). The MFI of phosphorylated mTOR protein (p-mTOR) did not change depending on the ratio of *NRAS*^Q61R^ (Fig. 4B-b). The MFI of p70S6K, one of the proteins downstream of mTORC1, in *NRAS*^Q61R^ HDLEC mixtures decreased significantly compared with that of *NRAS*^WT^ (p<0.001, p<0.001, and p=0.002, respectively) (S1 Fig b). The MFI of phosphorylated ERK (p-ERK) was significantly increased in *NRAS*^Q61R^ (p<0.001) (Fig 4B-c).” (P18, L304-314).

Regarding Fig. 4C-b, we revised the sentence to clarify that the MFI of p-mTOR after trametinib treatment was significantly decreased compared with the no treatment group (p<0.001) (Fig. 4C-b).

We revised the sentence “The MFI of AKT after trametinib treatment was significantly decreased compared with that of no treatment (p<0.001 and p<0.001, respectively) (Fig. 4C-b).” to “The MFI of p-mTOR after trametinib treatment was significantly decreased compared with that of no treatment (p<0.001) (Fig. 4C-b).” (P19, L315-318).

*- Please check the captions in the graphs of Figures 4B and 4C, they look confusing*

Thank you for your comments. We revised the figure legend of Fig 4B to “4B. Multiplex protein assay of p-Akt (a), p-mTOR (b), p-ERK (c), in *NRAS^Q61R^* and *NRAS*^WT^ HDLEC mixtures. Bars represent mean ± SD from triplicate wells. *p<0.05, **p<0.01, ***p<0.001, compared with 0%.” (P19, L323-326). We also added a figure legend for S1 Fig: “**S1 Fig. Multiplex protein assay of IRS-1 (a), p70S6K (b) in *NRAS^Q61R^* and *NRAS*^WT^ HDLEC mixtures.** Bars represent mean ± SD from triplicate wells. *p<0.05, **p<0.01, ***p<0.001, compared with 0%.” (P35, L601-603).

*- In the M&M section, the chapter “Analysis of NRAS mutational status” appears to be a refuse of the previous paper by the group, as NRAS mutational status was not analyzed in this study.*

Upon reviewing your comment, we agree that the title of this chapter may not accurately reflect the content of the current study. To address this, we revised the chapter title to "Insertion of the *NRAS* Mutation". (P6, L91), which more appropriately describes the procedures and analyses conducted in our study.

*Reviewer #3: In this manuscript, Yasue et al, aimed to explore a low-level somatic mutation in NRAS gene (c.182 A > G, Q61R) in LA patients. The authors generated stable lymphatic endothelial cells (LECs) with expressing NRASQ61R mutation using Lentiviral system to understand morphological and functional characterization, protein expression profiling, and metabolome analysis of those mutated cells compared to WT cells. They found out that NRASQ61R human dermal LECs showed poor tube formation and high cell proliferation and migration ability with increasing ratios of mutated cells. Moreover, signaling pathways analysis showed inactivation of the PIK3/AKT/mTOR pathway and hyperactivation of the RAS/MAPK/ERK pathway, which was improved by MEK inhibitor treatment. Overall, the findings they present are interesting, technically sound, and could be valuable to the field. Their claims are convincing and supported by the experimental data with comprehensive experimental controls. The manuscript provides relevant, up-to-date information and it is well written and supported with proper figures. All the data appears to be available within the manuscript and supplementary data.
I have minor suggestions that may be helpful for the reader to better understand the current study.*

1. *Figure 1: NRASQ61R HDLECs showed larger and irregular-shaped morphology compared with NRASWT HDLECs microscopically (Fig.1 a–c). Please define the image name which one is WT or Q61R? If there is no WT cell images, please include it as well.*

Thank you for the comment. We appreciate your suggestion to clearly define which images represent WT and Q61R cells. We have revised the second sentence of the Results as follows: “*NRAS*^Q61R^ HDLECs showed larger and irregular-shaped morphology compared with *NRAS*^WT^ HDLECs (Fig 1a and 1b) microscopically.” (P13, L215-216).

We also added the sentence “*NRAS*^WT^ and *NRAS*^Q61R^ HDLECs were mixed and cultured. Compared with *NRAS*^WT^ HDLECs, *NRAS*^Q61R^ HDLECs (fluorescent cells) were larger and their shapes were more irregular (Fig 1c).” (P13, L217-218). These revisions will ensure that the figure and accompanying text accurately and clearly convey the differences in morphology between WT and Q61R cells.

*2. Figure 2B: It is better to include X-axis name as (e.g. WT: Q61R cell ratio) in the figure for better understanding*

Thank you for the comment. We have added the X-axis name "Q61R cell ratio" to Figure 2B, parts a-c. This addition will make it clearer for readers to understand the data presented, particularly in relation to the ratio of Q61R cells in the context of our study.

2. *Figure 2D: It is better to include “DMSO treated control cells” to show the changing of the poor tube-forming ability*.

Thank you for your insightful comment regarding Figure 2D. Your suggestion to include "DMSO treated control cells" to illustrate the change in tube-forming ability is valuable. However, I would like to clarify that the results for the "no treatment" condition, which would be comparable with the no treatment cells, was already presented in Figure 2A. Consequently, to avoid redundancy and maintain focus on the specific changes induced by the treatments under study, we opted not to include the DMSO control in Figure 2D. We think that the results in Figure 2A adequately represent the baseline condition against which the effects of the treatments can be compared. We hope this explanation addresses your concern and provides clarity on our decision to structure the figures as we have.

*4. Figure 3B: Please define the Y-axis name in the graph (e.g. space ratio)*
Thank you for your comment regarding Figure 3B in our manuscript. We have added the title “space ratio” to the y-axis of Figure 3B to clarify what is being measured and presented.

*5. Figure 5: What is the control group?*

Thank you for your query regarding the control group in Figure 5. The control group used in the experiments shown in Figure 5 is, as detailed in our methods chapter titled "Suspension array" (P10, L163), the culture medium without any cells. This control was selected to provide a baseline for comparison, ensuring that any observed effects or changes could be attributed to the cellular interactions and conditions being studied, rather than to the medium itself.

*6. Page 26: “Among other amino acids, aspartic acid in NRASWT HDLECs was significantly higher than in NRASQ61R (p=0.008) (Fig.5)” Figure number should be 6.*

Thank you for bringing to our attention the incorrect figure reference in our manuscript. In response, we have carefully reviewed the manuscript and amended the text accordingly. The phrase "Fig 5" has now been corrected to "Fig 6" (P21, L366).
