# Supplementary material for: Changes in cell morphology and function induced by the NRAS Q61R mutation in lymphatic endothelial cells
Source: PLoS One. 2024 May 29;19(5):e0289187. doi: 10.1371/journal.pone.0289187 (PMC11135733; doi:10.1371/journal.pone.0289187)
Supplement: Attachment — Submitted filename: R2PONE-D-23-21611 comments.docx [file pone.0289187.s011.docx]

PONE-D-23-21611

Title: **Changes in cell morphology and function induced by the *NRAS* Q61R mutation in** **lymphatic endothelial cells**

***Journal Requirements:***

*Please review your reference list to ensure that it is complete and correct. If you have cited papers that have been retracted, please include the rationale for doing so in the manuscript text, or remove these references and replace them with relevant current references. Any changes to the reference list should be mentioned in the rebuttal letter that accompanies your revised manuscript. If you need to cite a retracted article, indicate the article’s retracted status in the References list and also include a citation and full reference for the retraction notice.*

We will review our reference list again to ensure its completeness and accuracy. We confirm that there are no retracted papers cited in our manuscript. Thank you for your guidance on this matter.

***Additional Editor Comments:***

*Authors have partially addressed the comments: The following points to be taken care before considering the article for publication.*

We thank the editor and reviewers for their helpful comments on our manuscript, which have contributed to its improvement. Our responses are outlined below. Changes to the text are in red font.

*Comments;*

1. *The Reviewer #2 still has some points which is critical.*

We have addressed the critical points raised by Reviewer #2 as follows. Thank you for bringing them to our attention.

*2. The following important RAS-related articles to be cited in the manuscript.*

*Importance of RAS in human cancer: PMID: 31255772; PMID: 27102293*

*How RAS can be modulated by miRNAs: PMID: 26620726*

*RAS mutations in other cancers: PMID: 22240207; PMID: 19628422*

Thank you for recommending these invaluable references on the importance of RAS in human cancer, its modulation by miRNAs, and RAS mutations in other cancers. We are grateful for your guidance and have duly cited these articles in our manuscript to enrich our discussion and support our findings. Your suggestions have significantly contributed to the depth and accuracy of our work.

“34. Murugan AK, Grieco M, Tsuchida N. RAS mutations in human cancers: Roles in precision medicine. Semin Cancer Biol. 2019 Dec;59:23-35. doi: 10.1016/j.semcancer.2019.06.007.

35. Tsuchida N, Murugan AK, Grieco M. Kirsten Ras* oncogene: significance of its discovery in human cancer research. Oncotarget. 2016 Jul 19;7(29):46717-46733. doi: 10.18632/oncotarget.8773.” (P34, L596- P35, L600)

*3. The spell, grammar and expressions to be checked as PLoS one does not edit for Grammar.*

Thank you for the reminder. We have reviewed the manuscript and corrected spelling, grammar, and expressions where necessary.

**Responses to Reviewer 2**

*The Authors have provided a revised version of the manuscript entitled “Changes in cell morphology and function induced by the NRAS Q61R mutation in lymphatic endothelial cells”. Although the Authors have addressed all the minor concerns and most of the major concerns that have been raised, some unaddressed points still persist:*

*- The mixed cultures in figure 1c are still difficult to evaluate as only NRASQ61R cells are visible. Since enhanced images of the NRASWT and NRASQ61R HDLECs with fluorescence have been added, consider deleting figure 1c.*

Thank you for your insightful comments and for highlighting the issues with Figure 1c in our manuscript. We appreciate your detailed review and agree with your assessment that the mixed cultures presented in Figure 1c are difficult to evaluate, because only NRASQ61R cells are prominently visible. As you suggested, we have removed Figure 1c from our manuscript.

We have made the necessary revisions to the text to reflect the removal of this figure and ensure that the manuscript remains cohesive and that the flow of information is not disrupted. We hope that this modification addresses your concern and improves the quality and readability of our manuscript.

*- Since no functional experiments have been performed demonstrating that hypersecretion of ANG-2 by NRASQ61R HDLECs leads to decreased VEGFR-3 levels, the work is still descriptive and provides only speculative conclusions.*

Thank you for your thoughtful feedback and for drawing attention to a crucial aspect of our study. We acknowledge the limitation you highlighted regarding the lack of direct functional experiments to demonstrate the impact of ANG-2 hypersecretion by NRASQ61R HDLECs on VEGFR-3 levels. As you correctly pointed out, our study of the relationship between ANG-2 hypersecretion and VEGFR-3 levels, as well as its subsequent effect on the maturation of peripheral LECs, is speculative and based on correlative evidence. Our statement was intended to propose a potential mechanistic insight based on the observed phenomena. In light of your feedback, we agree that it is imperative to clarify the speculative nature of these conclusions. Therefore, we revised our manuscript to include an explicit statement acknowledging the absence of direct functional experiments.

“Therefore, we hypothesized that the hypersecretion of ANG-2 from *NRAS*^Q61R^ HDLECs might decrease VEGFR-3 levels, associated with the inhibition of the maturation of peripheral LECs. However, this speculation requires additional functional analyses to confirm this relationship.” (P25, L427-429)

*- the same perplexity concerns the effect of inhibitors of the MEK and mTOR pathways which is not clearly linked to the NRAS mutation. It is unclear why mTOR inhibition has the same effects as MEK inhibition in cells without hyperactivation of the PI3K/AKT/mTOR pathway.*

Thank you for your insightful observation and for highlighting the complex dynamics between the *NRAS* mutation and effects of MEK and mTOR inhibitors. We acknowledge the significance of your concern and agree that the mechanisms underlying these observations are not fully understood at this stage of our research.

In our study, we observed that inhibitors targeting the MEK and mTOR pathways exhibited similar effects, even in cells where the PI3K/AKT/mTOR pathway was not hyperactivated. This finding raises important questions about the specificity and broader implications of these inhibitors in the context of NRAS mutations. We speculate that the convergence of the inhibitory effects of these pathways, despite the lack of overt hyperactivation of the PI3K/AKT/mTOR pathway, suggests a more complex, possibly compensatory, network of signaling interactions. However, we must emphasize that this interpretation is preliminary and necessitates further investigation. To address this gap in our understanding, we have added a section to our discussion highlighting the perplexity surrounding these effects and acknowledging the need for comprehensive functional studies. We think that elucidating these mechanisms will be crucial for developing more targeted and effective therapeutic strategies.

We have revised the text with reference to the points above.

“Our study findings did not fully clarify why mTOR and MEK inhibitors improved the tube formation and proliferation of patient-derived cells and mixed *NRAS*^Q61R^, as well as *NRAS*^WT^ HDLECs, or why only the MEK inhibitor prevented the proliferation of 100% *NRAS*^Q61R^ HDLECs and cell circularity. We suspect that these findings might indicate the presence of a complex and potentially compensatory signaling pathway. Therefore, comprehensive functional studies are necessary to elucidate these mechanisms.” (P24, L414-419)

We appreciate your highlighting of this issue, which underscores the complexity of cell signaling and the challenges in targeting these pathways. Your comment has been instrumental in guiding us to refine our manuscript and outline the directions for future research. Once again, we thank you for your constructive feedback and hope that our revisions and acknowledgment of these limitations reflect a thoughtful consideration of the complexities involved.

**Responses to Reviewer 3**

*Reviewer #3: All my comments have been addressed by the author. I have no more suggestions. The paper is ready for publishing.*

Thank you for your constructive feedback and for confirming that all your comments have been addressed satisfactorily. We appreciate your thorough review and are grateful for your support in deeming our paper ready for publication.
